# Prevalence and antimicrobial resistance of salmonellae isolated from eggs of local chicken in selected towns of Ethiopia

**DOI:** 10.1002/vms3.1529

**Published:** 2024-06-30

**Authors:** Belege Tadesse, Destaw Asfaw Ali

**Affiliations:** ^1^ Department of Veterinary Medicine School of Veterinary Medicine Wollo University Dessie Ethiopia; ^2^ Department of pathophysiology College of Veterinary Medicine and Animal Sciences University of Gondar Gondar Ethiopia

**Keywords:** antimicrobial resistance, Ethiopia, local chicken eggs, prevalence, *Salmonella*

## Abstract

**Background:**

Salmonellosis is one of the most common food‐borne diseases in industrialised and developing countries. In recent year, an increase in antimicrobial resistance among different *Salmonella* serotypes has been observed.

**Objective:**

A cross‐sectional study was conducted to assess the prevalence and antimicrobial susceptibility of *Salmonella* isolated from local chicken eggs in four selected towns in Ethiopia.

**Methods:**

A total of 115 eggs were examined to detect *Salmonella* by using standard microbiological methods. The susceptibilities of the isolates to nine antimicrobials were tested by the Kirby–Bauer disk diffusion method.

**Result:**

The study revealed that of the 115 eggs examined, 22 (19.1%) were positive for *Salmonella* of which 14 (12.2%) and 8 (7%) of the isolates were from shells and contents, respectively. The occurrence of *Salmonella* in egg shells and content and between different altitudes did not differ significantly (*p* > 0.05). Most isolates were resistant to more than three antimicrobials with a high resistance to kanamycin, ampicillin, nalidixic acid, cotrimoxazole, oxytetracycline and chloramphenicol.

**Conclusion:**

The results indicate the potential importance of local chicken eggs as source of multiple antimicrobial‐resistant salmonellae and the need for proper cooking before consumption. Further studies are required to describe the epidemiology of *Salmonella* in various agroclimatic zones of Ethiopia.

## Graphical abstract

The research showed moderate prevalence of salmonellosis (19.1%) from local chicken eggs and also high antimicrobial resistance to commonly used antimicrobials in the area

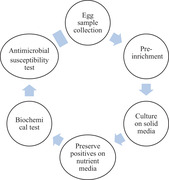

## 1 INTRODUCTION

Food‐borne diseases are among the most widespread global public health problems and their impact on the health and economy is high (Hendriksen et al., 2011). The worldwide increase of food‐borne infections with antibiotic resistant pathogens is of growing concern and is designated by the World Health Organization (WHO) as an emerging public health problem (CDC, 2006; Dagnew et al., 2020; Lynch et al., 2006; Much et al., 2007). Different studies revealed that *Salmonella* serotypes isolated from foods of animal origin have multidrug resistance with resistance to ≥3 antimicrobial classes (Molla et al., 2003; Sarba et al., 2020). Globally, *Salmonella* exhibits extensive resistance profiles, which have been associated with higher rates of morbidity and mortality and costs associated with disease (Angulo et al., 2004). It is also one of the most prevalent zoonotic bacteria (Velusamy et al., 2010).

Since the beginning of the 1990s, strains of *Salmonella* which are resistant to a range of antimicrobials, including first choice agents for the treatment of humans, have emerged and are threatening to become a serious public health problem (CDC, 2006; Fey et al., 2003; Kimura et al., 2005; Threlfall et al., 2000). This increased emergence and widespread of antimicrobial‐resistant *Salmonella* strains in chickens and other food animals and humans may be associated with the use of medicated feeds in intensive animal husbandry systems, subtherapeutic doses and indiscriminate uses of antimicrobials both in animal and human treatments (Molla et al., 2003; Tufa et al., 2018; Van et al., 2007; Walsh & Fanning, 2008). In recent year, *Salmonella* has been reported from several food‐related items and the spread of antimicrobial‐resistant *Salmonella* strains have become a serious health hazard in different countries including Ethiopia (Ejo et al., 2016; Ketema et al., 2018; Prestinaci et al., 2015). The resistance of *Salmonella* to commonly used antimicrobials in both public health and veterinary sectors in Ethiopia is also increased (Eguale, 2018; Eguale et al., 2015; Ketema et al., 2018).

Contamination with *Salmonella* in poultry products can occur at multiple steps along the food chain, which includes production, processing, distribution, retail marketing, handling and preparation (Cui et al., 2005; Much et al., 2007).

Consumption of raw contaminated eggs is a common source of food‐borne diseases (Cox et al., 2000). *Salmonella enterica* serotype Enteritidis is transmitted to the human food supply through eggs of hens that appear healthy (Porwollik et al., 2005). Egg contents may be contaminated with *Salmonella* by two routes: trans‐ovarian (vertical transmission) or trans‐shell (horizontal transmission) (Cox et al., 2000; FAO/WHO, 2002). In trans‐ovarian transmission, *Salmonella* are introduced from infected reproductive tissues to eggs prior to shell formation while in horizontal transmission the bacteria invade the egg through the shell after the egg is laid (ACMCF, 2001). *Salmonella* serotypes associated with poultry reproductive tissues includes *Salmonella* Enteritidis, *S*. Typhimurium and *S*. Heidelberg. Among these, *S*. Enteritidis may have better invasive properties and, therefore, found more frequently in reproductive tissues (ACMCF, 2001).

Infected hens can deposit *Salmonella* in either the yolk or albumen of developing eggs because of the colonisation of different regions of the reproductive tract (Bichler et al., 1996; Gast et al., 2007). Moreover, freshly laid eggs are typically reported to contain no more than a few hundred *Salmonella* cells (Chen et al., 2002; Gast & Holt, 2000a). Therefore, prompt refrigeration to internal temperatures of 7.2°C or lower protects consumers by preventing bacterial multiplication to more dangerous levels inside eggs during storage (Chen et al., 2002).

Despite some studies on the prevalence of *Salmonella* in Ethiopia, mainly in pig, cattle, poultry meat, minced beef (Dagnew et al., 2020; Ejeta et al, 2004; Molla & Mesfin, 2003; Nyeleti et al., 2000) and humans (Nyeleti et al., 2000), the status of the salmonellae and its antimicrobial resistance in eggs of local chicken is still not sufficiently addressed. However, studies carried out elsewhere indicated that chicken eggs are important sources of *Salmonella* particularly among those raw consumers (FSIS, 2005). There is also a report on the existence of multidrug‐resistant *Salmonella* strains in chicken farms in Ethiopia (Bekele & Ashenafi, 2010). Therefore, the objectives of this study were to estimate the prevalence and antibiotic resistance pattern of salmonellae isolated from eggs of local chicken in four selected towns in Ethiopia.

## 2 MATERIALS AND METHODS

### 2.1 Study area

The study was conducted in three towns of Oromia region, namely, Bishoftu, Modjo, and Dukem and one town of Addis Ababa city administration, namely, Akaki, from December 2013 to May 2014. Bishoftu is situated 47 km South East of Addis Ababa in East Shewa, Oromia region, Ethiopia. It is located at 8°44’N and 38°58’E at an elevation of 1880 m above sea level (m.a.s.l). It has an average annual rainfall of 1151.6 mm with a bimodal distribution. The long rainy season ranges from July to September and contributes for about 84% of the total rainfall. The dry season lasts from October to February. The mean annual minimum and maximum temperatures are 12.3°C and 27.7°C, respectively, with an average annual temperature of 20°C and a mean relative humidity of 61.3% (CSA, 2008).

Modjo, the second site, is found in the Central Ethiopia, Eastern Shoa zone of the Oromia region, 70 km southeast of Addis Ababa. This town exists at latitude of 8°35’N and longitude of 39°10’ E at an elevation of 1777 m.a.s.l. The average minimum temperature and maximum are 18°C and28°C, respectively (NMSAE, 2012).

Akaki, the third site, is located in Addis Ababa, the capital city of Ethiopia at 9°1’N and 38°44’E at an average altitude of 2500 m.a.s.l. The area experiences a bimodal rainfall pattern with short rainy season from March to May and the long rainy season from June to September. The annual rainfall is about 800–1100 mm with a mean annual humidity of 61.5%. The mean annual minimum and maximum temperature is about 8.5°C and 30.7°C, respectively (NMSAE, 2012).

Dukem, the last site of study, is located in Oromia Regional State, Oromia Special Zone at a distance of 37 km from Addis Ababa at 08°48′N and 38°54′E and at an elevation of 1950 m.a.s.l. The area has a mean annual rainfall of 907 mm and mean annual minimum and maximum temperature of 17.2°C and 20.5°C, respectively, with an average annual temperature of 18.7°C (NMSA, 2008).

### 2.2 Study design, sampling and sample size determination

The study was a cross‐sectional, and sample size was calculated by using the formula *n* = *Z* ^2^ *p* (1 – *p*)/*d* ^2^ according to Thrusfield (2007) with an expected prevalence (*p*) of 8% (Assefa et al., 2011), a 95% confidence interval and an absolute precision (*d*) of 0.05. A total of 115 whole noncracked eggs produced from local chickens were purchased from different persons from a market. All the sampled local chicken eggs were produced from the backyard free‐range production system. One egg from one egg seller was collected. Eggs were individually packed with sterile polyethylene plastic bag and transported to the Microbiology laboratory.

### 2.3 Isolation and identification

#### 2.3.1 Culture methods

The isolation and identification of the bacteria was according to the method described by the International Organization for Standardization (ISO 6579:2002). The culture methods included a pre‐enrichment stage, selective enrichment and plating on selective media.

##### Pre‐enrichment

The bags containing eggs were opened in the laboratory and broken eggs were rejected. Sterile cotton swabs dipped in sterile 10 mL buffered peptone water (BPW) (HiMedia Laboratories Pvt. Ltd, Mumbai, India) were used to swab the entire surface area of egg shells. The cotton swabs were put in screw capped bottles containing BPW and mixed by stirring with the swab. To collect egg contents, the egg surfaces were sterilised by immersing in 75% alcohol (ethanol) for 2 min and air‐dried in drying cabinets for 10 min. The eggs were then aseptically cracked with a sterile scalpel and the contents were collected in sterile universal bottles and mixed thoroughly by stirring with glass rods or spoon or using a vortex for 30 s. Twenty‐ five millilitres of the mixed egg contents were taken and inoculated into 225 mL of BPW and homogenised for 2 min with a shaker. The shells and contents were incubated at 37°C for 24 h.

##### Selective enrichment

After pre‐enrichment 0.1 mL of the broth was transferred in to a tube containing 10 mL of Rappaport‐Vassiliadis (RV) broth (HiMedia Laboratories Pvt. Ltd, Mumbai, India) and another 1 mL was transferred in to a tube containing 10 mL of Selenite F broth (Titan Biotech Ltd, Rajasthan, India). The inoculated RV broth was incubated at 42°C for 24 h and the inoculated Selenite F broth at 37°C for 24 h. Positive broth showed a grey‐white and turbid zone extending out from the inoculated drop in RV and Selenite F broths, respectively.

##### Plating on selective media

Xylose lysine desoxycholate (XLD) agar (HiMedia Laboratories Pvt. Ltd, Mumbai, India) and brilliant green agar (BGA) (Titan Biotech Ltd, Rajasthan, India) plates were used for plating out and identification purpose. A loopful of inoculums from the RV and Selenite F broths were transferred and streaked on to the surfaces of XLD and BGA agars separately. The plates were incubated at 37°C for 24–48 h. After incubation, the plates were examined for the presence of colonies, which on XLD agar are pink with a black centre whereas lactose‐positive salmonellae are yellow with or without blackening. Typical colonies of *Salmonella* on BGA are pink, 1–2 mm in diameter, and cause the colour of the medium to change to red. Three to five typical and /or suspected colonies were picked from the selective plating media and streaked on to nutrient agar (Oxoid CM0003, Basingstoke, England) and incubated at 37°C for 24 h.

#### 2.3.2 Biochemical tests

The colonies plated on nutrient agar were inoculated in to tubes containing the following; triple sugar iron (TSI) agar (Oxoid), lysine decarboxylase agar (Oxoid), urea broth (HiMedia), tryptone broth (HiMedia), MR‐VP broth (HiMedia) and incubated for 24 or 48 h at 37°C. Colonies producing an alkaline slant with acid (yellow colour) but on TSI with hydrogen sulphide production, positive for lysine (purple colour), negative for urea hydrolysis (orange colour), negative for tryptophan utilisation (indole test) (yellow‐brown ring) and negative for Voges–Proskauer (no colour change of the medium/colourless), were considered to be *Salmonella* (ISO 6569:2002).

### 2.4 Antimicrobial sensitivity tests

Twenty‐two isolates were tested for antimicrobial susceptibility. The antimicrobial susceptibility testing was done by using the Kirby–Bauer agar disk diffusion method as described in Clinical and Laboratory Standards Institute (CLSI) (2020) on Mueller–Hinton agar (Oxoid Ltd., UK). Briefly from each biochemically positive sample, 3–5 well isolated colonies were used in the test for antimicrobial sensitivity. The top of each colony was touched with a loop, and transferred in to a tube containing 4 mL brain heart infusion broth (Oxoid, Canada) and incubated for 6–8 h at 37°C. The broth was either directly adjusted to the 0.5 McFarland standards or incubated at 37°C until it achieved the turbidity of the 0.5 McFarland standards. The dried surface of the Mueller–Hinton agar plate was then inoculated by streaking the swab over the entire agar surface. The procedure was done under a laminar flow to avoid contamination.

The following antimicrobial disks (Oxoid Ltd., UK) were applied on the plates: ampicillin (10 µg), chloramphenicol (30 µg), kanamycin (30 µg), streptomycin (25 µg), nalidixic acid (30 µg), oxytetracycline (30 µg), cotrimoxazole (25 µg), ceftriaxone (30 µg) and amoxicillin (30 µg). Six antimicrobial disks were placed on the seeded agar surface of 15×150 mm Petri dishes, separated from each other so as to avoid overlapping of inhibition zones. The plates were placed in an incubator at 37°C and examined after 16–18 h of incubation. The diameters of the zones of complete inhibition were measured using sliding callipers. The zones of inhibition were interpreted by referring to zone diameter interpretive standards of CLSI (2020) for *Enterobacteriaceae*, and organisms are reported as susceptible, intermediate or resistant to the tested agents. Isolates were grouped as multi drug resistant if they are resistant to three or more than three antimicrobials (Magiorakos, 2012).

### 2.5 Statistical analysis

Data were entered and managed in Microsoft Office Excel 2007 and analysis was done using SPSS version 20. Descriptive Statistics was used to describe the data. Associations were tested by the Chi square test and Alpha was set at 0.05.

## 3 RESULT

### 3.1 Prevalence of *Salmonella*

The results on the occurrence of *Salmonella* in egg shells and contents are presented in Table 1. Of the 115 examined eggs, 22 (19.1%) were positive for *Salmonella*. None of the samples were positive for both egg shells and egg contents. The prevalence of *Salmonella* in egg shells and contents were 12.2% and 7%, respectively, and did not differ significantly (*p* > 0.05) (i.e. *X* ^2^ = 1.192; *p* = 0.275). Altitude (i.e. location) did not significantly affect the occurrence of *Salmonella* (*X* ^2^ = 1.57; df = 2; *p* = 0. 457).

**TABLE 1:** Prevalence of *Salmonella* by sample types and source (location) of samples.

Sample source	Number examined	Shell positive No. (%)	Content positive No. (%)	SoC^a^ positive No. (%)	SaC^b^ positive No. (%)
Bishoftu	28	4 (14.3%)	0 (0.00%)	4 (14.3%)	0
Modjo	30	5 (16.7%)	5 (16.7%)	10 (33.3%)	0
Akaki	29	4 (13.8)	1 (3.4%)	5 (17.2%)	0
Dukem	28	1 (3.6%)	2 (7.1%)	3 (10.7%)	0
Total	115	14 (12.2%)	8 (7%)	22 (19.1%)	0

^a^ Egg shell or content.

^b^Egg shell and content.

### 3.2 Antimicrobial resistance

#### 3.2.1 Mono drug resistance

The monodrug resistance features of the isolates are given in Table 2. All isolates were resistant to kanamycin and ampicillin. More than half of the isolates were resistant to each of the tested antimicrobials except to ceftriaxone and streptomycin. Resistance to oxytetracycline, ceftriaxone and nalidixic acid was observed in 19, 7 and 21 isolates, respectively. A significant number of isolates (12/22) displayed intermediate resistance to ceftriaxone.

**TABLE 2:** Numbers of susceptible, moderately susceptible (intermediate) and resistant isolates by antimicrobials and antimicrobial class.

Antimicrobials	Antimicrobial class	Sensitive	Intermediate	Resistant
Amoxicillin	Penicillin	4	1	17
Ampicillin	Penicillin	0	0	22
Ceftriaxone	Cephalosporins	3	12	7
Chloramphenicol	Amphiphenicols	3	1	18
Cotrimoxazole	Trimethoprim	1	2	19
Kanamycin	Aminoglycoside	0	0	22
Nalidixic acid	Quinolones	1	0	21
Oxytetracycline	Tetracycline	2	1	19
Streptomycin	Aminoglycoside	9	2	11

#### 3.2.2 Multiple drug resistance

The multidrug resistance profiles of the isolates are depicted in Table 3. All isolates were resistant to more than three antimicrobials and 15 resistance profiles were recorded. Twenty isolates were resistant to more than six antimicrobials. The K, OT, NA, C, AMC, AMP, CT phenotype was recorded in four isolates followed by the K, OT, NA, C, S, AMC, AMP, CT and K, OT, CRO, NA, C, AMP, CT phenotypes. Ten of the MDR isolates were resistant to ceftriaxone and nalidixic acid.

**TABLE 3:** Multiple antimicrobial resistance patterns of *Salmonella* isolates.

Number of antimicrobials	Resistance pattern	No. of isolates	No. of MDR
Four	K, OT, AMC, AMP,	1	1
	K, OT CRO, AMP, CT	2	3
Five	K, NA, S, AMC, AMP	1	
Six	K, OT, NA,AMC, AMP, CT	1	1
Seven	K, OT,NA, S, C, AMP, CT	2	12
	K, CRO, NA, C, AMC, AMP, CT	1	
	K, OT,CRO,NA,C,AMP,CT	3	
	K, OT, NA, C, AMC, AMP, CT	4	
	K, OT, NA, C, S, AMC, AMP	1	
	K, OT, RO, NA, AMC, AMP, CT	1	
Eight	K, OT, NA, C, S, AMC, AMP, CT	3	6
	K, OT, CRO, NA, C, S, AMP, CT	1	
	K, CRO, NA, C, S, AMC, AMP, CT	1	
	K, OT, CRO, NA, C, AMC, AMP, CT	1	
Nine	K, OT, CRO, NA, C, S, AMC, AMP, CT	2	2

AMP, ampicillin; C, chloramphenicol; K, kanamycin; S, streptomycin; OT, oxytetracycline; NA, nalidixic acid; CT, cotrimoxazole; CRO, ceftriazone; AMC, amoxicillin.

## 4 DISCUSSION

In this study, the overall prevalence of *Salmonella* was 19.1%. This finding is higher than the report of Assefa et al. (2011) who reported an overall prevalence of 11.5% in Kombolcha, Ethiopia. Lower prevalence ranging from 0% (Humphrey, 1994) to 7% (Baumler et al., 2000) in the United Kingdom and 11.8% in Yemen (Rihab et al., 2010) were also reported. Singh et al. (2010) also reported relatively lower level of incidence in the chicken eggs collected from poultry farms and marketing channels in North India. The prevalence of *Salmonella* from fumigated egg shells was reported as 0% (Assefa et al., 2011) and 1.2% (Gholam et al., 2009). To reduce penetration of bacteria in to content to a minimum level, it is advisable to fumigate the eggs as soon as they are collected and while they are still warm (Gholam et al., 2009). The probable reason for the high level of occurrence of *Salmonella* in the current study may be due to high prevalence of *Salmonella* in the study towns and lack of proper housing for layers.

The prevalence of *Salmonella* in eggs shells (12.2%) in this study was higher than reported from Kombolcha (6.3%), Ethiopia (Assefa et al., 2011) and elsewhere ranged from 0.04% to 20% (Adesiyun et al., 2005; Food Standards Agency, 2004; Harsha et al., 2011; Little et al., 2006; Murchie et al., 2007; Suresh et al., 2005; Suresh et al., 2006). The high *Salmonella* prevalence in eggs shells in the present study when compared to the above findings could probably be due to heavy infection in the layers, environmental contamination, in effective cleaning and disinfecting of materials used for egg handling and storage (Davies & Breslin, 2003).

The 7% *Salmonella* prevalence from egg content was comparable with the 7.7% prevalence recorded at Kombolcha (6.8%), Ethiopia (Assefa et al., 2011), and the 9.2% prevalence in Trinidad and Tobago (Adesiyun et al., 2005). Lower prevalence estimates were reported in the United Kingdom (Little et al., 2008) and in New Zealand (FSIS, 2005). Other studies from Ireland, United Kingdom and the United States reported negative for *Salmonella* in egg contents (Food Standards Agency, 2004; Murchie et al., 2007). The deposition of *Salmonella* is more often on the yolk membrane than in the interior contents of the yolk (Gast & Holt, 2001). *Salmonella* can survive in the albumen but is inhibited from growing for an extended period of time due to high pH and inhibitory factors in the albumen (Gast & Holt, 2000b). On the contrary, yolk is a rich microbial medium with little capability of inhibiting *Salmonella*. Rapid growth of *Salmonella*, especially at storage temperature above 20°C can occur (Braun & Fehlhaber, 1995; Humphrey & Whitehead, 1993).

In this study, all isolates were resistant to ampicillin. This finding is in agreement with previous reports in Ethiopia (Addis et al., 2011), South India (Suresh et al., 2006), Nigeria (Akinyemia et al., 2005) and Cameroon (Akoachere et al., 2006) where more than 90% of the isolates were resistant to ampicillin. Abdi et al. (2017) also reported that 97.8% of the *Salmonella* isolates from poultry farms in Southern Ethiopia were resistance to ampicillin.

Resistance to amoxicillin, ceftriazone, cotrimoxazole and oxytetracycline observed in this study is higher than reported by Murugkar et al. (2005) in north‐eastern India. Resistance to cotrimoxazole among poultry isolates was reported from Senegal (Bada‐Alambedji et al., 2006), Mexico (Zaidi et al., 2006) and the United States (Zhao et al., 2006). The high rate of resistance to oxytetracycline among *Salmonella* isolates is in agreement with other studies on *Salmonella* isolates from food animals in Ethiopia (Abdi et al., 2017; Eguale et al., 2014). Oxytetracycline has been frequently used for treatment and prophylaxis in the current study area (Eguale et al., 2016). The frequent use of this antimicrobial could have contributed to the development of resistant to tetracycline.

Almost all isolates (96%) were resistant to NA. Higher level of resistance were also reported elsewhere: 100% in Iran (Fallahi et al., 2013) and 89% in France (Cailhol et al., 2005). In a survey by Fardsanei et al. (2020) in Tehran and Shahpouri and Eghbalzade (2009) in Zanjan, resistance to NA was reported in 90.6% and 57.7% of the isolates, respectively. Although the higher occurrence of quinolone‐resistant isolates in several countries could be explained in terms of the irrational use of the drugs in livestock and humans, the factors associated with the higher occurrence of nalidixic acid‐resistant isolates in Ethiopia are unclear.

The proportion of isolates resistant for three or more of antimicrobials was higher than other studies conducted in Ethiopia in apparently healthy slaughtered cattle and food items (Alemayehu et al., 2003; Zewdu & Cornelius, 2009). In addition, the antimicrobial susceptibility patterns of the *Salmonella* isolates indicated that a large proportion of the isolates were resistant to a variety of drugs that included oxytetracycline, cotrimoxazole, ampicillin, nalidixic acid and kanamycin. These findings are comparable with results of an earlier report on *Salmonella* isolated from poultry carcasses and giblets in Ethiopia (Tibaijuka et al., 2002). According to Gyles (2008), the frequencies and patterns of antimicrobial resistance in *Salmonella* vary depending on time, region, serotypes, the particular farm (source), layers versus broilers and the antimicrobial agent.

Besides the increasing resistance to commonly used antibiotics in animals and humans, there is a concurrent increase in multiple‐resistant *Salmonella* isolates worldwide (Carraminana et al., 2004; Eguale, 2018; Molla et al., 2003). In a study on 54 *Salmonella* strains isolated from a total of 301 chicken meat and giblets in Ethiopia, 31 isolates were resistant to one or more antimicrobials of which 17 (54.8%) exhibited multiple resistance to up to six different antimicrobials (Tibaijuka et al., 2002).

All salmonellae isolated in the current study were resistant to more than three antimicrobials. This indicates that eggs need special care before consumption. The consumption of uncooked eggs favours the transmission of drug‐resistant salmonellae. The spread of antimicrobial resistance *Salmonella* in the developing world is very common (Swanenburg et al., 2001) and the dissemination and emergence of multidrug resistance *Salmonella* could be because of the indiscriminate use of antimicrobials (Chandra et al., 2006; Onyango et al., 2008). The occurrence of resistance to three or more antimicrobials in the majority of the isolates in the current study agrees with the report of Eguale (2018). The reason for the high rate of MDR might be attributed to the level of use of specific antibacterial agents in the poultry farms, which affected the level of selection pressure that contributed to maintaining resistance genes in the bacterial population (Marshall & Levy, 2011).

The current study reports important information on the prevalence and antimicrobial resistance features of salmonellae isolated from eggs of local chicken in selected towns of Ethiopia. But the identification of the serotype was not done, which is the limitation of the study.

## 5 CONCLUSION AND RECOMMENDATIONS

The study demonstrated that *Salmonella* is a prevalent pathogen in local chicken. Eggs from local chicken are highly contaminated and potential sources of food‐borne Salmonellosis. A considerable proportion of the eggs harbour multidrug‐resistant *Salmonella* and are serious public health risks. It is recommended that eggs should be kept at lower temperatures and cooked properly before consumption. Further studies are required to describe the risk factors associated with the occurrence of *Salmonella* in local chicken in different agroclimatic zones of Ethiopia and the factors that potentially contribute to the emergence of drug‐resistant *Salmonella*.

## AUTHOR CONTRIBUTIONS

Belege Tadesse (principal author): Conceptualisation, investigation, resources, methodology, formal analysis, writing – review and editing. Destaw Asfaw Ali: Data curation, formal analysis, resources, methodology, writing – original draft.

## CONFLICT OF INTEREST STATEMENT

The authors declare that they have no conflicts of interest. They also declare that the information presented herein their work is original, and all sources of materials used for the research work have been duly acknowledged.

## FUNDING

This research was supported by the grant fund of Addis Ababa University.

### ETHICAL STATEMENT

Not applicable.

### PEER REVIEW

The peer review history for this article is available at https://publons.com/publon/10.1002/vms3.1529.

## Data Availability

The data used to support the findings of this study are included within the article in the prevalence table and also can be acquired from the principal author upon reasonable request.
